# Advanced fluorescence lifetime-enhanced multiplexed nanoscopy of cells

**DOI:** 10.1515/mim-2024-0029

**Published:** 2025-01-30

**Authors:** Samrat Basak, Roman Tsukanov

**Affiliations:** Third Institute of Physics—Biophysics, Georg August University, 37077 Göttingen, Germany; Department of Chemistry and Center for NanoScience, Ludwig-Maximilians-Universität München, 81377 Munich, Germany

**Keywords:** fluorescence lifetime imaging microscopy, super-resolution microscopy, single-molecule localization microscopy, DNA-PAINT, multiplexed imaging, metal-induced energy transfer

## Abstract

In this review paper, we summarize the significant advancements in the field of fluorescence lifetime imaging microscopy (FLIM), particularly wide-field FLIM with single-molecule sensitivity, achieved using the time-correlated single-photon counting-based position-sensitive LINCam system. Fluorescence lifetime adds valuable information beyond conventional intensity-based imaging, enabling diverse applications across research fields. Here, we focus on three primary bioimaging applications: (I) single-molecule FLIM in the far-red spectral region, (II) fast and multiplexed super-resolution imaging of cells, and (III) three-dimensional super-resolution imaging with high axial localization precision. Recent advances in position-sensitive detector technologies offer exciting opportunities for high-throughput super-resolution imaging with enhanced localization precision.

## 1 Introduction

Fluorescence lifetime imaging microscopy (FLIM) has revolutionized bioimaging by introducing a layer of information that surpasses the capabilities of conventional intensity-based imaging methods. By leveraging fluorescence lifetime to characterize molecular environment and interactions with unparalleled robustness and precision, FLIM offers a powerful means to differentiate between spectrally overlapping signals. These features position FLIM as an indispensable tool for deciphering complex biological processes, enabling molecular-level insights unattainable with traditional microscopy techniques [1]. For instance, the innovative HaloTag technology developed by the Kai Johnsson group has enabled lifetime-based multiplexed imaging of live cells [2]. Additionally, FLIM can utilize the autofluorescence of NADH and FAD for real-time imaging of cellular metabolism. By detecting subtle changes in the fluorescence lifetimes of these molecules, FLIM monitors shifts in cellular states, responses to environmental changes, and disease markers, providing a non-invasive assessment of cellular health [3].

This review highlights the latest advances in wide-field FLIM, including FLIM with single-molecule sensitivity (smFLIM). Recent technological developments have propelled wide-field FLIM forward, enabling significant progress in the field [4], [5]. Noteworthy examples include: (1) fast FLIM imaging achieved through single-objective light-sheet microscopy paired with a time-resolved SPAD array detector [6]; (2) rapid and accurate smFLIM using a commercial time-gated single-photon camera [7]; (3) wide-field electro-optic fluorescence lifetime imaging microscopy (EO-FLIM), which enables kilohertz frame-rate imaging and has been applied to studying neuronal activity in adult Drosophila [8]; and (4) a novel wide-field FLIM approach that splits detection photons between an emCCD camera for emitter position localization and an SPAD for lifetime detection [9].

Wide-field smFLIM enables rapid imaging across large fields of view, showcasing the critical role of lifetime information in describing complex cellular structures and interactions. In this context, we discuss the implementation of wide-field smFLIM using the TCSPC-based LINCam system, which combines high accuracy in lifetime determination with exceptional sensitivity and robustness [10]. The LINCam system presents a compelling alternative to commonly used Confocal Laser Scanning Microscopy (CLSM). Unlike CLSM, which relies on high detector quantum yield and pinhole-based background light rejection to achieve high localization precision, the LINCam system supports rapid full-frame acquisition. It also offers flexibility in selection of an excitation illumination modes by switching between different illumination schemes, including conventional wide-field illumination, Highly Inclined and Laminated Optical sheet (HILO) illumination, and Total Internal Reflection (TIR) illumination. Notably, TIR illumination enhances contrast while providing easy and robust alignment, making it ideal for high-throughput smFLIM imaging with improved signal-to-background ratio. This review outlines the LINCam system’s capabilities, requirements, and imaging protocols, illustrating how wide-field smFLIM can complement and, in some cases, surpass the performance of CLSM-based smFLIM systems.

The paper’s primary focus is the integration of FLIM into super-resolution microscopy techniques, particularly Single-Molecule Localization Microscopy (SMLM) [11], [12]. Lifetime information significantly enhances the capabilities of SMLM, offering researchers exciting opportunities for fast multiplexed imaging, environmental sensing, and advanced 3D SMLM imaging with high axial resolution via methods such as metal-induced energy transfer (MIET) [13] and graphene-induced energy transfer (GIET) [14], [15].

By incorporating lifetime-based imaging within advanced imaging techniques such as CLSM and wide-field smFLIM, we demonstrate how these methods enhance multiplexing capacity and overcome limitations associated with traditional multi-color imaging, including spectral overlap and chromatic aberration. smFLIM enables high-resolution, rapid, and multiplexed imaging across diverse biological systems. Moreover, smFLIM’s compatibility with super-resolution techniques, particularly within the FL-SMLM framework, facilitates unprecedented exploration of molecular interactions, cellular metabolism, and dynamic biological processes. Altogether, these advancements establish FLIM as a transformative bioimaging tool, enabling researchers to delve deeper into the intricacies of biological systems.

## 2 Materials and methods

### 2.1 LINCam system specifications

**Figure 1:**
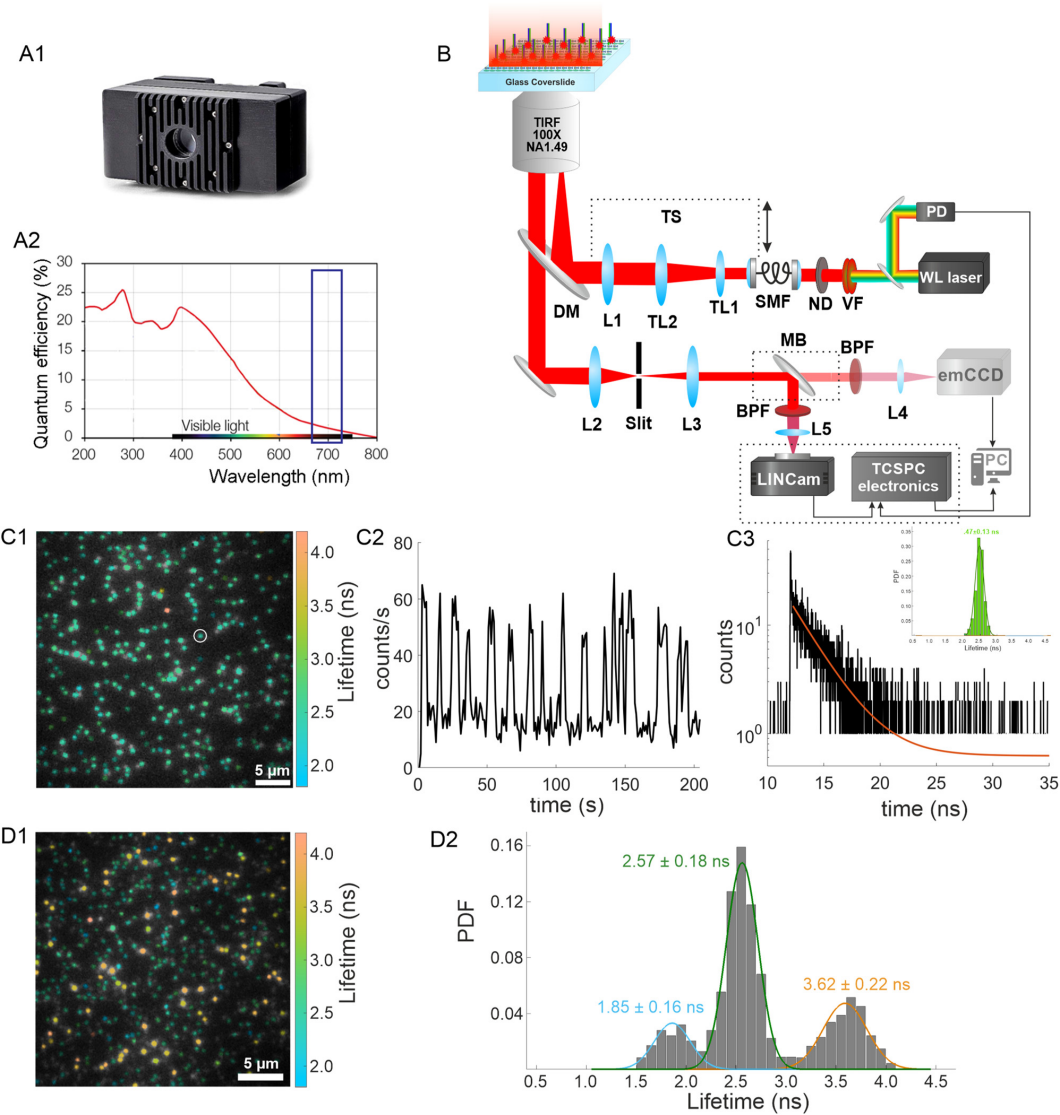
Wide-field smFLIM imaging with TCSPC-based position sensitive LINCam system. (A1) Picture of the LINCam system detector alongside its quantum efficiency plot (A2), with the far-red spectral region highlighted by a purple rectangle. (B) Schematic representation of the custom-built optical setup. (C1) smFLIM image of single Atto 655 molecules conjugated to DNA and immobilized on a surface. (C2) Intensity time trajectory of the single molecule highlighted by a white circle in (C1). (C3) TCSPC histogram and single-exponential fit of the same molecule shown in (C1). Inset: Lifetime histogram of all molecules detected within the field of view. (D1) smFLIM image of a mixture of Cy5, Atto 655, and Atto 647N fluorophores. (D2) Cumulative lifetime histogram of the mixed sample shown in (D1). The data is fitted with three Gaussian functions, with the corresponding average lifetime values and standard deviations displayed next to the peaks. Adapted from Reference [16].

LINCam is a TCSPC-based position-sensitive photon-counting system that detects photons one by one in counting mode. Unlike conventional time-only detectors, such as SPADs or hybrid detectors, LINCam also captures the position of each detected photon. The system employs a microchannel plate-based photomultiplier tube (MCP-PMT) as its light-sensitive component, see Figure 1(A1). This tube is a vacuum-sealed assembly comprising a photocathode, an MCP stack, and a position-sensitive anode [10]. The photocathode converts incoming photons into photoelectrons, which are then amplified by the MCPs. The resulting electron avalanche, consisting of several million electrons, is read out by spatially separated electrodes. These electrodes enable the reconstruction of the incident photon’s position with a spatial accuracy of tens of microns and a temporal accuracy of ∼50 ps. The type of photocathode determines the system’s spectral sensitivity. For the LINCam system S20, the quantum yield is below 20 % for the visible spectrum, decreasing from 20 % in the blue to ∼3 % in the far-red spectral region, as shown in Figure 1(A2). However, the camera confidently detects single fluorophores even in the most challenging far-red spectral region thanks to the extremely low background noise, which results in a high signal-to-background ratio (high sensitivity), see Figure 1(C1–C3) [16]. The only source of background noise resulting in “false” photon detections is thermal noise, which is typically below 50 Hz for the entire S20 sensor (25 mm in diameter). For photocathodes sensitive to the far-red spectral region (such as HiQE Red), thermal noise may reach 500 Hz. However, this is counterbalanced by the enhanced signal detection capability of up to 1 MHz, delivering an excellent signal-to-background ratio. The dynamic range of the LINCam system reaches 1 M counts/s, with optimal photon detection efficiency in the range of 250–500 k counts/s [17].

### 2.2 Wide-field single-molecule FLIM using LINCam system

In general, the TCSPC-based LINCam system can be integrated into most commercial microscopes. This also includes different techniques, for example spinning disk confocal microscopy (SDCM) or light sheet microscopy. Naturally, the system can also be incorporated into different types of custom-built optical systems, as shown in Figure 1(B) and described in detail below, or a hybrid of commercial and custom-built systems. However, while designing a wide-field smFLIM system, the following guidelines should be taken into account: (1) for practical considerations, it is convenient to use the LINCam system not as a standalone imaging system, but in tandem with a conventional intensity-based scientific camera with large dynamic range. Due to the high sensitivity of the LINCam system, first imaging step includes sample characterization using intensity-based camera, in order to spot a region of interest (ROI) and optimal imaging conditions. Then, the emission light shall be directed to the LINCam system to perform smFLIM imaging. (2) Incorporation an adjustable slit (1D or 2D) into emission path of an optical system. This enables for physical restriction of the emission light to the selected ROI, while avoiding from the undesired light to reach the LINCam system. (3) Leveraging the advantages of different excitation illumination schemes available in wide-field microscopy: by changing the excitation beam incidence angle, one can enhance the contrast while minimizing reflections in an emission path. Such reflections might appear as peaks in the TCSPC curve, which could affect the quality of the TCSPC histogram fit. (4) Achievement of minimal background light environment in the optical room for smFLIM microscopy. Due to the high sensitivity of the detector, undesired background light has to be minimized. Optimally, the smFLIM system shell be positioned in a separate optical room.

### 2.3 Laser sources for TCSPC-based position sensitive LINCam system

In TCSPC-based smFLIM systems, pulsed lasers are required. The pulse duration has to be short (below 100 ps) compared to the measured lifetime. The latter typically spans within range of 1–6 ns for fluorescent proteins and synthetic dyes used for biomedical imaging. Therefore, depending on the particular lifetimes of dyes used for FLIM measurement, the optimal laser repetition rate can vary between 25 and 80 MHz, which corresponds to a time window in range 12.5–40 ns, accommodating 5–10× fluorophore lifetime (*τ*). Careful selection of laser repetition rate is crucial for accurate lifetime measurement. Either pulsed diode lasers with a fixed output wavelength or the white-light laser source can be employed. The diode lasers typically provide higher output power, as compared to white light laser sources, while the output wavelength of the latter can be flexibly changed by acousto-optic tunable filters (AOTFs). Alternatively, filters with variable bandwidth or optical band pass filters with fixed bandwidth can be employed for the most efficient excitation of a fluorophore of choice. The suitable laser source has to be selected for particular smFLIM application.

On the technical side, all the electronics of the LINCam system have to be interconnected and synced. For the particular case, a laser serves as a master device triggering the LINCam system with positive or negative NIM signals (100 mA @ 50 Ω). The quality of the pulse is important for precise offset timing. Therefore, in case the laser output signal is not sharp enough, an optical triggering of the TCSPC-based camera can be employed, as detailed below.

### 2.4 Wide-field single-molecule FLIM measurement with LINCam system

Remarkably, it has been shown that different types of single fluorophores with overlapping emission spectra can be accurately identified based on their distinct lifetimes, with minimal crosstalk, see Figure 1(D1–2). Such separation was demonstrated using a mix of DNA-conjugated fluorophores Cy5, Atto 655, and Atto 647N commonly used in the bioimaging field. The molecules were specifically immobilized to glass surface via biotin-avidin chemistry. Single molecules were detected and its fluorescence lifetimes were determined by TCSPC histograms tail-fitting with subnanosecond precision. The use of fluorophore on/off states detection algorithm efficiently minimized the background signal by taking into account mostly informative photons, then improving signal-to-background ratio and, as a result, accuracy in lifetime determination. Such optimized lifetime data analysis made separation of three different fluorophores feasible even in the most challenging for single-molecule detection far-red spectral region. Thanks to high signal-to-background ratio, identification of blinking on and off states is straightforward, making the single-molecule localization microscopy application feasible for wide-field smFLIM. The development of wide-field smFLIM using TCSPC-based position sensitive detectors represents a significant milestone in smFLIM imaging. This approach effectively addresses longstanding challenges in fluorescence microscopy by combining high signal-to-background ratio, fast acquisition speed, and high precision in lifetime determination.

### 2.5 Description of custom-built wide-field smFLIM setup

Our implementation of wide-field smFLIM system is based on a custom-built optical setup as shown in Figure 1(A) and Reference [16]. The pulsed super-continuum white light laser (WL Laser) (SuperK Fianium, NKT Photonics) served as an excitation source with variable pulse repetition rates. Variable filter (VF) (SuperK Varia, NKT Photonics) was used to flexibly adjust the spectral range of the excitation output light. Neutral density filters (ND) (NE10A-A, NE20A-A, Thorlabs) in tandem with the variable neutral density filter (NDC-50C-4-A, Thorlabs) were used to adjust the laser excitation power. A custom-made photodiode (PD) was employed to pickup the pulses optically and trigger the LINCam system. Then, the laser beam was coupled into a single-mode optical fiber (SMF) (P1-460B-FC-2, Thorlabs) with a typical coupling efficiency of ∼30 %. On the optical fiber output, the decoupled collimated laser beam was expanded by a factor of 3.6× using telescope lenses (TL1 and TL2). Then, the beam was focused onto the back focal plane of the TIRF objective (UAPON 100× oil, 1.49 NA, Olympus) using achromatic lens (L1) (AC508-180-AB, Thorlabs). The optical setup allowed for switching between different excitation illumination schemes (EPI, HILO, and TIR) by mechanical shifting of the excitation beam with respect to the optical axis by moving a translation stage (TS) (LNR25/M, Thorlabs). The smooth lateral translation of a sample was achieved by using a high-performance two-axis linear stage (M-406, Newport), while the focusing was achieved by sliding the objective attached via custom-made clamp to an independent one-dimensional translation stage (LNR25/M, Thorlabs) equipped with a differential micrometer screw (DRV3, Thorlabs). The spectral separation of the collected fluorescence light from the excitation pathway was achieved using a multi-band dichroic mirror (DM) (Di03 R405/488/532/635, Semrock), which directed the emitted fluorescence light towards the tube lens (L2) (AC254-200-A-ML, Thorlabs). The field of view was physically restricted in the emission path by an adjustable slit aperture (SP60, OWIS) positioned in the image plane. Lenses (L3) (AC254-100-A, Thorlabs) and (L4) (AC508-150-A-ML, Thorlabs) re-imaged the emitted fluorescence light form the slit onto an emCCD camera (iXon Ultra 897, Andor) or, alternatively onto a LINCam system. The switching between the two imaging systems was achieved by placing additional mirror (MB) in order to redirect the emission light into another arm of the emission path, while the lens (L5) (AC508-250-A-ML, Thorlabs) was used to create an image on the LINCam system. Band-pass filters (BP) (BrightLine FF 536/40, BrightLine FF 609/62, BrightLine HC 692/40, Semrock) were used to further block the scattered excitation light. The total magnification of the optical system on the emCCD camera was 166×, resulting in an effective pixel size in the sample space of 103.5 nm. The total magnification for imaging with TCSPC-based LINCam system was 222×, resulting with the virtual partitioning of the field of view into 512 × 512 pixels in the effective pixel size of 191.6 nm in the sample space. The temperature was kept at 22 ± 1 °C to ensure the mechanical stability of the optical setup.

## 3 Results

### 3.1 Fluorescence lifetime single-molecule localization microscopy

Single-molecule localization microscopy (SMLM) is a family of powerful super-resolution techniques based on a blinking behavior of individual fluorescent molecules. These blinking events are computationally localized to determine the emitter’s precise position. All the localisations are then summed up and used to generate a super-resolution image [11]. Fluorescence lifetime single-molecule localization microscopy (FL-SMLM) is a powerful SMLM variant that builds upon incorporating fluorescence lifetime information as an addition to conventionally used intensity-based SMLM. At its core, FL-SMLM enables precise localization of individual fluorophores, achieving resolutions well below the diffraction limit. Over thousands of blinking cycles of each single emitter, FL-SMLM accumulates localized fluorophore positions and its lifetimes and reconstruct super-resolved FLIM image at unprecedented detail. In FL-SMLM, high achievable localization precision is complemented by adding fluorescence lifetime data, which enables lifetime-based identification of fluorophores even within densely labeled regions. Fluorophores for FL-SMLM imaging are selected not only based on their blinking performance and brightness, but also for their distinct lifetime values and narrow lifetime distributions, allowing for lifetime-based separation of targets emitting in the same spectral region. The powerful combination of spatial and lifetime information significantly leverages FL-SMLM’s ability to identify multiple targets within a single spectral channel, avoiding chromatic aberration and enhancing multiplexing capacity for bioimaging applications. Recent advancements in the microscopy techniques have further expanded FL-SMLM’s potential. For instance, combining confocal FL-SMLM with Image Scanning Microscopy (ISM) [18], has shown a 1.3-fold improvement in lateral localization precision, offering an efficient pathway to enhance resolution without additional experimental complexity. Additionally, lifetime information can be used to precisely localize emitter in axial direction via Graphene-Induced Energy Transfer (GIET) [14], [15] or Metal-Induced Energy Transfer (MIET) [13], [19]. By combining SMLM-based techniques such as dSTORM and DNA-PAINT with GIET/MIET, it is possible to reach isotropic three-dimensional localization with nanometer precision [20], [21]. This combination is particularly beneficial for studying complex biological structures located close to a coverslip interface in 3D, as GIET/MIET techniques provide extreme axial localization precision. In addition, FL-SMLM has proven valuable in dynamic studies where temporal resolution is critical. In summary, FL-SMLM is emerging as a powerful approach for studying molecular interactions and structural dynamics in biological systems. By combining high spatial precision with lifetime information, multiplexing, and enhanced 3D imaging capabilities, FL-SMLM offers a comprehensive toolbox for exploring complex cellular arrangements with unprecedented detail.

### 3.2 Fast and multiplexed super-resolution imaging via fluorescence lifetime DNA-PAINT

**Figure 2:**
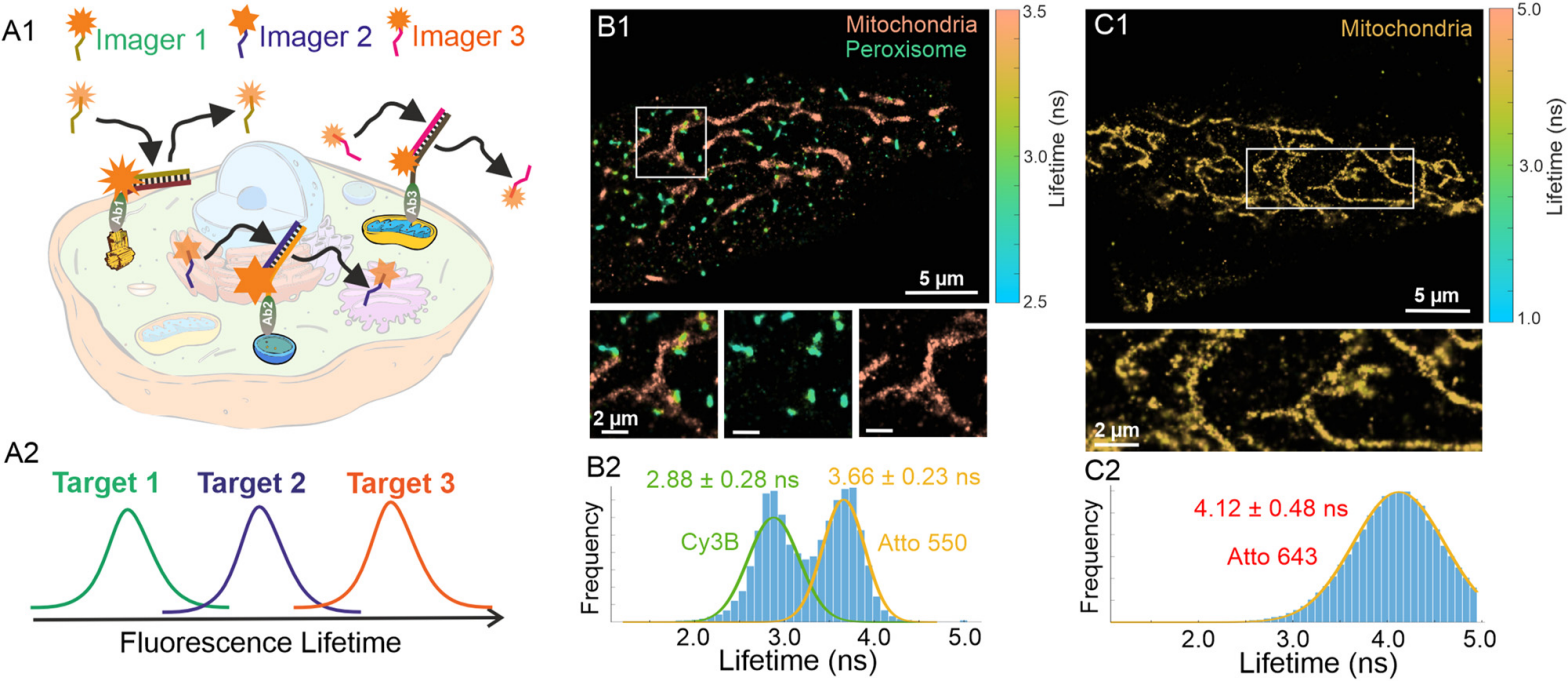
Fluorescence lifetime DNA-PAINT for parallelized multiplexed imaging of a cell. (A1) Principle of FL-PAINT: three targets are labeled with transiently binding imagers carrying fluorophores that emit in the same spectral region but have distinct fluorescence lifetimes. (A2) The targets are imaged simultaneously and identified based on lifetime information, as illustrated in the schematic. (B1) Wide-field FL-PAINT image of mitochondria and peroxisomes in a HeLa cell, labeled with Cy3B and Atto 550, which emit in the orange spectral region. Zoomed-in views of the region highlighted by the white box are shown at the bottom. (B2) lifetime histogram demonstrating target identification based on lifetime information for the image in (B1). (C1) FL-PAINT image of mitochondria in a HeLa cell labeled with Atto 643, emitting in the far-red spectral region. (C2) lifetime histogram corresponding to the FL-PAINT image shown in (C1). Adapted from Reference [28].

DNA-PAINT is a powerful super-resolution microscopy technique belonging to the SMLM family of techniques, capable of achieving molecular resolution while circumventing the photobleaching effect. DNA-PAINT relies on a transient binding of a short single-stranded DNA (imager) to its complementary docking strands affixed to the imaged target [22]. DNA-PAINT is particularly suitable for multiplexed imaging, as different targets of interest can be labeled with docking strands having different DNA sequences orthogonal to each other, then completely avoiding the crosstalk between targets [23]. Remarkably, imaging of all targets can be accomplished using the same fluorophore via sequential imaging of targets using technique called Exchange-PAINT, then avoiding the chromatic abberation completely [24], [25]. Despite unlimited multiplexing capacity, Exchange-PAINT imaging can be time consuming, as the total acquisition time scales linearly with the number of targets. Furthermore, extensive washing steps are required. Furthermore, such solutions exchange can affect sample integrity and result in undesired mechanical drift. Fluorescence Lifetime DNA-PAINT (FL-PAINT) [17] is a versatile variant of DNA-PAINT designed for fast and multiplexed imaging through parallel acquisition of targets, then shortening acquisition time and washing steps. Using the concept of FL-SMLM, in FL-PAINT the targets are labeled with fluorophores emitting in the same spectral range but distinct in their lifetimes, see Figure 2(A1–2). FL-PAINT fully relies on the striking advantages of DNA-PAINT: no photobleaching effect, multiplexing capacity of DNA-PAINT, designability of binding/unbinding rates, high specificity of labeling and minimal crosstalk between imaged targets. In addition, DNA-PAINT is fully flexible in the selection of fluorophores, which is highly important for FL-PAINT imaging and allows for careful selection of fluorophores that are both compatible with DNA-PAINT (non-sticky, narrow brightness distributions) and have distinct lifetimes then allowing for targets separation with minimal crosstalk. Furthermore, tight control over relative concentrations of imagers in FL-PAINT experiment allows for careful adjustment of number of binding events for each imaged target, enabling for equalization of number of binding events for different targets with optimal stoichiometry.

Unlike Exchange-PAINT, FL-PAINT imaging circumvents the need for fluid exchange and enhances throughput of an experiment. This is particularly important for imaging sensitive biological systems whose integrity can be affected by extensive buffer exchange. The optimal (to date) combination of fluorophores reported for FL-PAINT imaging in the orange spectral range is Alexa Fluor 555, Cy3B, and Atto 550, see Figure 2(B1–2), while the optimal fluorophores for FL-PAINT imaging in far-red spectral range are Alexa Fluor 647 and Atto 643, see Figure 2(C1–2). For the analysis of FL-PAINT data, we utilized the custom Matlab-based TrackNTrace Analysis Package (Lifetime Edition) [17], [26], which is readily accessible online via link: https://github.com/scstein/TrackNTrace/releases/tag/v2.0. Also, FL-PAINT applications for neuronal imaging has been demonstrated [27]. FL-PAINT imaging in blue and green spectral regions is less advantageous as typical biological samples (cells and tissues) have autofluorescence in these spectral regions, which directly affects imaging quality and introduces artifacts. Nonetheless, FL-PAINT imaging in these spectral regions is possible with careful control measurements and imaging protocol optimization. We note that constant progress in the development of synthetic labels and fluorophores with properties important for FL-PAINT (brightness, distinct lifetimes with narrow lifetime distributions) promises exciting enhancement of FL-PAINT imaging with even higher acquisition speed, improved localization precision and simultaneous imaging of three and even more targets in a single imaging round. Furthermore, FL-PAINT compatibility with other multiplexing approaches like Exchange-PAINT or spectral demixing promises great potential for a high throughput multiplexed imaging with minimal sample disturbance.

#### 3.2.1 Comprehensive comparison between wide-field and confocal FL-PAINT

FL-PAINT was implemented using both wide-field and confocal smFLIM optical setups. These two approaches are complementary to each other; however, a head-to-head comparison is the best way to explore the real capabilities of the both systems [28]. The LINCam system is robust and does not require sensitive alignment of optics, while CLSM-based smFLIM systems are sensitive and rely on the alignment of an optical setup, ensuring precise overlap of excitation and detection volumes to achieve optimal fluorescence signal detection. However, confocal systems have several features that make them advantageous in certain experimental situations: high localization precision is required (ensured by the high quantum yield of avalanche photodiodes (APDs) typically used with CLSM); physical rejection of out-of-focus light thanks to a pinhole in the emission path; deep sample imaging is feasible; 3D imaging through optical sectioning is also possible. Additionally, confocal systems equipped with APD detectors possessing higher quantum efficiency in the far-red spectral region as compared to the LINCam system. Such versatility makes FL-PAINT suitable for a broad spectrum of bioimaging investigations, ranging from rapid multiplexed imaging of cells and tissues to potential future clinical applications.

### 3.3 Lifetime-assisted 3D super-resolution imaging with high axial localization precision

**Figure 3:**
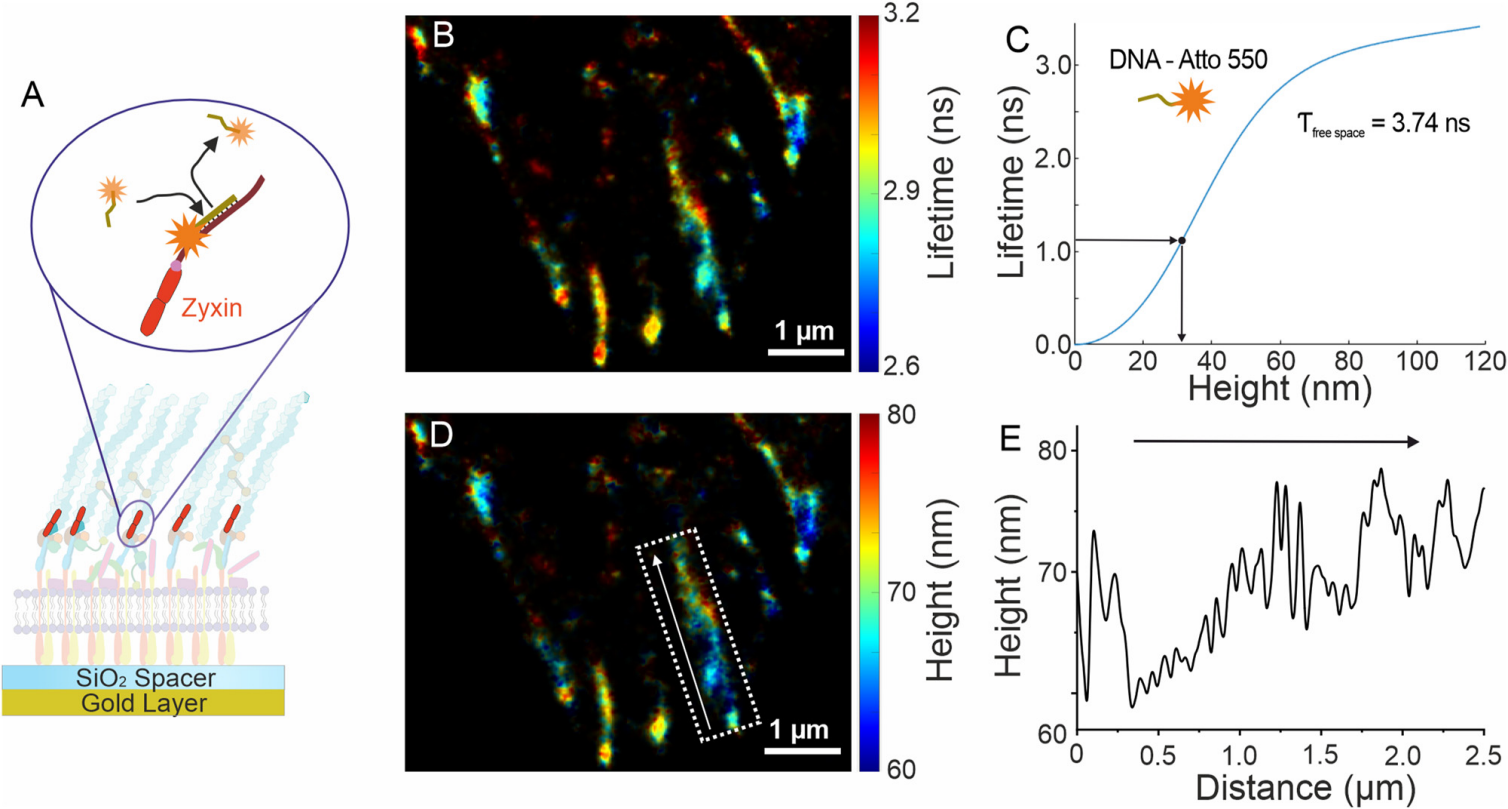
Lifetime-assisted 3D super-resolution imaging. (A) MIET-PAINT imaging of the focal adhesion complex (FAC), illustrating the MIET-PAINT principle and sample geometry. (B) Wide-field MIET-PAINT lifetime image of the Zyxin protein within the FAC. (C) Theoretical lifetime-height dependency (MIET curve) for the Atto 550 fluorophore. (D) 3D MIET-PAINT image reconstructed from the lifetime image in (B) using the MIET curve shown in (C). (E) Height profile of the Zyxin cluster highlighted by the white box in (D), with the arrow indicating the direction of the height profile.

Fluorescence lifetime information can be used not only for environmental sensing and multiplexed imaging but also for precise localization of emitters in the axial direction. For the purpose, metal-induced energy transfer (MIET) [13] and graphene-induced energy transfer (GIET) [14], [15] can be employed. In case of MIET, the energy transfer occurs between an emitter (donor) and plasmons inside thin metal layer (acceptor). Using the lifetime-height dependency derived from MIET theory [29], experimentally measured emitter lifetimes are converted into heights, then transferring two-dimensional FLIM into three-dimensional image. A powerful combination of SMLM and MIET exploits the high resolution achieved by SMLM techniques in the lateral directions with the high axial resolution achieved by MIET, reaching isotropic resolution of up to nanometer scale. For example, dSTORM combined with MIET was shown to achieve isotropic localization precision in 3D [20]. Particularly high localization precision in axial direction can be achieved by MIET for fluorophores exhibiting a high photon budget, long fluorescence lifetimes, and single-exponential behavior of the photon arrival times histogram. Unlike dSTORM, which can be used with a limited selection of dyes showing optimal blinking pattern, DNA-PAINT can be flexibly used with most of fluorophores, then making it particularly suitable for MIET imaging with highest axial localization precision. Moreover, DNA-PAINT offers unlimited multiplexing capacity, making it a perfect choice for multiplexed 3D super-resolution bioimaging. The concept of MIET-PAINT has been demonstrated by performing multiplexed imaging of Focal Adhesion Complex (FAC) in 3D, see Figure 3 (A) [30]. The sample consisted of cells positioned on thin gold surface topped with SiO_2_ spacer layer. DNA-PAINT imaging was performed using the LINCam system and the super-resolved FLIM image was obtained, see Figure 3(B). Using MIET curve specifically calculated for the fluorophore of choice, as shown in Figure 3(C), the FLIM image was converted into 3D MIET-PAINT image, as shown in Figure 3(D). By analyzing height profiles of Zyxin, it was possible to explore its arrangement in 3D, on a single FAC level. For this purpose, a specific FAC cluster can be selected, and a height profile of Zyxin can be obtained and analyzed, see Figure 3(E). Similar to the analysis of FL-PAINT data, MIET-PAINT data was processed using the Matlab-based TrackNTrace package (Lifetime Edition) [26], [30], which is available online at the following link: https://github.com/scstein/TrackNTrace/releases/tag/v2.0. MIET-PAINT imaging of the same cell for additional targets is possible via Exchange-PAINT protocol, revealing details regarding colocalization and function of FAC. On the experimental side, wide-field smFLIM approach allows to image using TIR illumination, then significantly enhancing the signal-to-background ratio, which was particularly important for MIET-PAINT imaging, due to massive presence of fluorescently labeled imager in the solution which elevates the background signal. The full potential of MIET-PAINT for multiplexed imaging of complex biological systems is yet to be explored by the new generation of wide-field lifetime detectors and implementation of the concept for confocal smFLIM systems.

## 4 Discussion

Wide-field smFLIM equipped with the LINCam system is a powerful tool which can be used for broad range of advanced bioimaging applications: environmental sensing, lifetime-based multiplexed super-resolution imaging via FL-SMLM, 3D imaging with high axial resolution via metal-induced energy transfer. It takes an initial effort to integrate and adapt the LINCam system to perform with a specific optical setup or microscope. For example, one has to make sure that two ports in a microscope are available for imaging, the first port for conventional scientific camera used for initial sample characterization, while the second is reserved for the LINCam system. The LINCam imaging system has to be light-tight: no light from outside should penetrate the imaging systems. In addition, back reflections of the excitation light shell be minimized. Once imaging system adaptation is accomplished, wide-field smFLIM performs robustly and reliably, becoming a workhorse of FLIM imaging with single-molecule sensitivity. Remarkable ease of use and operation, as well as compatibility with most commercial microscopes, leverage its potential for the broader imaging community. In a gold rush for highly multiplexed bioimaging, LINCam system is a very attractive option thanks to the precision in position and lifetime determination, ease of use, and an output format, which allows post-experimental division into virtual pixels and selection of a time bin.

## 5 Conclusions and outlook

The applications and results highlighted in this view obtained with LINCam systems equipped with the S20 sensors [31], featuring relatively low quantum efficiency. Recently, significant progress in development of a new generation of sensors with significantly higher quantum efficiency was achieved, for example Photonis HiQE [32]. Implementing wide-field FLIM imaging with the new generation of detector leverages imaging quality in a far-red spectral region, extremely challenging for S20 sensors [28]. Such advancements significantly enhance and democratize smFLIM, FL-SMLM as well as its applications. Also, FL-SMLM imaging with higher localization precision becomes feasible, potentially reaching the performance of confocal FL-SMLM. Such smFLIM systems will find even broader range of applications, from chemistry and time-resolved microscopy/spectroscopy to astronomy and quantum optics. The core obstacle for the broad spread of LINCam systems can be its relatively high price tag, as compared to emCCD/sCMOS scientific cameras. However, along with enhanced performance and wider range of applications available to the new generation of lifetime cameras, as well as growing interest in a wide-field smFLIM in the imaging community, its future is bright. The price of the LINCam system will be reduced as it becomes a commercially mass-produced product, making it available to a broader circle of new users from various research fields.
